# Exome sequencing in patients with chronic central serous chorioretinopathy

**DOI:** 10.1038/s41598-019-43152-3

**Published:** 2019-04-29

**Authors:** Rosa L. Schellevis, Myrte B. Breukink, Christian Gilissen, Camiel J. F. Boon, Carel B. Hoyng, Eiko K. de Jong, Anneke I. den Hollander

**Affiliations:** 1 Department of Ophthalmology, Donders Institute of Brain, Cognition, and Behaviour, Radboud University Medical Center, Nijmegen, The Netherlands; 2 Department of Genetics, Donders Institute of Brain, Cognition, and Behaviour, Radboud University Medical Center, Nijmegen, The Netherlands; 3 Department of Ophthalmology, Leiden University Medical Center, Leiden, The Netherlands

**Keywords:** Genome informatics, Genome-wide association studies, Medical genomics, Medical genetics, Retinal diseases

## Abstract

Chronic central serous chorioretinopathy (cCSC) is a multifactorial eye disease characterized by subretinal fluid accumulation that leads to vision loss. Clinically, cCSC is associated with stress, hypercortisolism and corticosteroid use, and is more frequent in males (80%) than in females (20%). Current genetic studies on cCSC have thus far focussed on common variants, but familial occurrence of cCSC also suggests a role for rare variants in the disease susceptibility. Therefore, in this study, we performed exome sequencing of cCSC patients to elucidate the role of rare (protein-altering) variants in the disease. Exome sequencing was performed on 269 cCSC patients and 1,586 controls. Data were processed according to the Genome-Analysis-Toolkit (GATK) best practices. Principal component analysis was performed to check for genetic ancestry and only unrelated subjects of European descent were retained. Burden, SKAT and SKAT-O tests were performed using 2 different grouping criteria. One group included protein-altering variants only, while the other contained synonymous and splice site variants as well. The gene-based analyses were performed using the SKAT R-package correcting for two principal components using two approaches; (1) on the entire cohort correcting for sex and (2) on males and females separately. Additionally, the gene-based associations of genes at previously reported cCSC loci were investigated. After filtering, the dataset contained 263 cCSC patients (208 males [79%]) and 1352 controls (671 males [50%]) carrying 197,915 protein-altering variants in 16,370 genes and 330,689 exonic variants in 18,173 genes. Analysis stratified by sex identified significant associations with the *PIGZ* (P_SKAT_ = 9.19 × 10^−7^ & P_SKAT-O_ = 2.48 × 10^−6^), *DUOX1* (P_SKAT_ = 1.03 × 10^−6^), *RSAD1* (P_SKAT_ = 1.92 × 10^−7^ & P_SKAT-O_ = 8.57 × 10^−8^) and *LAMB3* (P_Burden_ = 1.40 × 10^−6^ & P_SKAT-O_ = 1.14 × 10^−6^) genes in female cCSC patients, after correction for multiple testing. The number of rare variant carriers in these genes was significantly higher in the female cCSC cohort compared to female controls (45,5% vs. 18.5%, P = 1.92 × 10^−6^, OR = 3.67 [95% CI = 2.09–6.46]). No significant associations were identified in the entire cohort nor in the male patients. In this exome study on cCSC patients, we have identified *PIGZ*, *DUOX1, RSAD1* and *LAMB3* as potential new candidate genes for cCSC in females. The sex-specific associations identified here suggest a possible interaction between rare genetic factors and sex for cCSC, but replication of these findings in additional cohorts of cCSC patients is necessary.

## Introduction

Central serous chorioretinopathy (CSC) is a vision-threatening eye disease characterized by serous fluid accumulation between the neuroretina and retinal pigment epithelium (RPE), supposedly secondary to a dysfunctional choroid and outer blood-retina barrier of the RPE^1^. The incidence of CSC is estimated to be 1.7 in 100,000 in women and 9.9 in 100,000 in men with most cohorts confirming this ~80% male predominance^1,2^. Two distinct forms of CSC have been described, distinguished by the duration of symptoms and extend of the RPE alterations. Acute CSC usually resolves spontaneously within ~3 months, whereas in chronic CSC (cCSC) fluid accumulation persists and more widespread RPE atrophy occurs^1^. The etiology of CSC is unknown, but associations with stress, hypertension and the use of corticosteroids have been described^1^. A genetic component for the disease was suggested because of familial occurrence of CSC^3,4^, and so far a limited number of genetic studies have been performed for cCSC.

Candidate gene studies have described associations between cCSC and common single nucleotide polymorphisms (SNPs) in the *CFH, ARMS2, CDH5* and *NR3C2* genes^5–8^, as well as copy number variations (CNVs) of the *C4B* gene^9^. Additionally, the first genome-wide association study on cCSC confirmed the role of *CFH* in the disease^10^. Other genes suggested to be involved in the genetics of cCSC include *CD46, CFHR1, CFHR4, KCNT2, TNFRS10A* and *VIPR2*^10,11^. So far, only common variants have been associated with cCSC, however, familial clustering of cCSC has also hinted towards a possible role for rare variants in disease susceptibility^3,4^.

Therefore, to elucidate the role of rare (protein-altering) variants in cCSC we performed exome sequencing on 269 cCSC patients. We performed single-variant association and gene-based tests on the entire cohort and by stratifying the analyses for sex. Additionally, we investigated the gene-based associations of genes at loci that have previously been associated with cCSC. Here, we report new potential candidate genes for cCSC in females, and further broaden our knowledge on the disease etiology.

## Materials and Methods

### Library preparation and sequencing

#### cCSC patients

In this study, 269 cCSC patients who visited the outpatient clinic of the Department of Ophthalmology of the Radboud university medical center, Nijmegen, the Netherlands were included. Patients were diagnosed with cCSC based on multimodal retinal imaging as described previously^6,10^. DNA of cCSC patients, isolated from blood, was purified with the QIAamp DNA purification kit (Qiagen), according to manufacturer’s instructions. 3 µg of purified DNA was used as input for the SureSelect^XT^ target enrichment system for Illumina paired-end multiplexed sequencing (Version B4, August 2015, Agilent Technologies).

Library preparation was performed according to the manufacturer’s instructions. Samples were hybridized with the SureSelect All Exon V4 (Agilent Technologies) capture library and post-hybridization addition of the index tags was performed on the Dynabeads MyOne Streptavidin T1 beads (ThermoFisher Scientific). Midway and final quality checks were performed with Tapestation high sensitivity D1000 screentape (Agilent Technologies) and a Qubit dsDNA high sensitivity assay (ThermoFisher Scientific). A total of 8 samples was pooled for sequencing in one lane. Sequencing was performed at the Department of Genetics of Maastricht University Medical Center+, Maastricht, The Netherlands, using an Illumina HiSeq2000 with 2*100 bp chemistry.

### Controls

As population controls, 1586 parents that were part of a large cohort of intellectual disability trios were used^12^; no ophthalmologic imaging was available for these individuals. Control samples were sequenced with the Agilent SureSelect V4 enrichment kit in a diagnostic setting at the Beijng Genomics Institute (BGI) in Copenhagen between August 2013 and March 2015. Sequencing was performed on an Illumina HiSeq instrument with 101-bp paired-end reads^12^. The median coverage of the exomes of both cCSC patients and controls was 75X.

This study was performed according to the guidelines of the Declaration of Helsinki and was approved by the local ethics committee Commissie Mensgebonden Onderzoek (CMO) of the region Arnhem-Nijmegen. Written informed consent was obtained for all subjects involved in the study.

### Variant calling

Bam to FastQC extraction was performed for both controls and cCSC patient data with Picard-tools (v 1.90). Files of patients and controls were processed together to minimize batch effects^13^. Duplicate reads were marked with Picard-tools and reads were aligned to the reference genome (GRCh37.p5 with alternate haplotypes excluded) using BWA-MEM (version v.0.7.12). Sequencing data was processed according to the Genome-Analysis-Toolkit (GATK) best practices (v3.8). Briefly, base recalibration was performed using the following known sites: dbsnp version 144, Mills and 1000 G gold standard and 1000 G phase1 high confidence insertion/deletions (INDELs). Variant calling was performed with haplotypecaller in g.vcf mode specified on the SureSelect V4 regions flanked with 200 bp. Individual g.vcfs were merged into batches of 200 samples and joint genotyping on the whole cohort was performed per chromosome using genotypeGVCFs with the newQual option. Sites with a minimal average coverage of 15X in both the cCSC patients and control datasets were retained for downstream analyses.

### Variant quality score recalibration (VQSR)

Variant recalibration was performed with GATK (v3.8) using the recommended settings^13^. For SNPs, Hapmap3.3 and OmniExpress 2.5 chip sites were used as truth and training sets, while the 1000 G phase1 data was used for training only. For INDELs, the Mills and 1000 G gold dataset was used as truth and training sets. For both SNPs and INDELs “dbsnp v138 excluding sites after dbsnp v129 sites” were used to indicate known variants. The following arguments were used during the generation of the variant recalibration model for SNPs: Quality by Depth (QD), Mapping Quality (MQ), MQRankSum, ReadPosRankSum, Fisher strand (FS), Strand odds ratio (SOR), InbreedingCoeff, and MQCap = 60. For INDELs the MQ option was removed, as recommended. The variant recalibration model was applied to the dataset for SNPs and INDELs separately and the data was filtered based on the 99.5 and 99 sensitivity tranche, respectively.

### Population stratification, Cryptic relatedness and Sex-check

All variants overlapping with the Hapmap3 data were extracted and combined with the original Hapmap3 data containing individuals of various genetic ancestries. The merged dataset was pruned using PLINK (v1.90b3y), with a window size of 50 kb, stepsize of 5 kb and variant inflation factor of 2. Principal component analysis (PCA) was performed on the data to account for population admixture. The first 2 PCA components were plotted with R (v 3.2.0), and ancestry labels were added to the Hapmap data. After filtering only individuals from European descent were retained (Fig. S1 in Document S1).

To assess cryptic relatedness between individuals in the dataset, kinship coefficients were calculated with KING (v2.0)^14^. Proportion of zero identity by state (IBS) was plotted against the estimated kinship coefficient with R (v 3.2.0) (Fig. S2 in Document S1). Samples were removed that had a kinship coefficient indicative of being either first degree (0.177–0.354), second degree (0.177–0.0884) or third degree (0.0884–0.0442) relatives. Finally, reported sex was compared with the data available for the X chromosome using the sex-check option in PLINK, with a threshold of F < 0.35 for women and F > 0.7 for men.

### Variant filtering

Variants located in low complexity regions of the genome were removed^15^. Multiallelic variants were extracted with VCFtools (v0.1.13) and split using the splitMultiallelic and LeftAlingandTrimVariants option in GATK (v3.8). After splitting, biallelic and multiallelic SNPs and INDELs were combined and tested for Hardy-Weinberg equilibrium P > 1 × 10^−8^ and a minor allele count of 1. Variants from the adult-onset cancer genes captured in the American College of Medical Genetics and Genomics recommendations for incidental findings (*BRCA1, BRCA2, MLH1, MSH2, MSH6, PMS2, MUTYH*) were removed to reduce the risk of secondary findings^16^. Remaining variants were annotated with TabAnno (https://github.com/zhanxw/anno). In order to perform the downstream gene analysis, two different groupfiles were made using a Python script (available on request). The protein-altering variant groupfile contained nonsynonymous, stop gain/loss, start gain/loss, codon gain/loss, frameshift and essential splice site variants, while the exonic groupfile included both protein-altering variants and synonymous, exon and variants in the complete splice site.

### Statistical analysis

Data was converted to binary PLINK files and imported into the SKAT R-package (v. 1.2.1)^17,18^. As the SKAT package does not offer a function to analyze a large set of single variants, one large “dummy” geneset was generated containing all the variants in the input file. The association of each variant was calculated with a loop through the “dummy” geneset (Script File S1). The SKAT R-package uses the efficient resampling method to also include extremely rare variants in the analysis^17^. The SKAT null model adjusted for small sample size was performed for a binary phenotype correcting for two principal components and sex (for the entire cohort) with the resampling method “bootstrap.fast”. The generated file was also used to calculate the variant weights based on minor allele frequency using the “Get_Logistic_Weights” function of the SKAT package (v. 1.2.1)^17,18^.

Next, a minor allele frequency (MAF) weighted Burden, SKAT, and SKAT-O analysis was performed with the “SKATBinary.SSD.All” option. These analyses were performed on either the entire cohort or the cohort stratified by sex using the protein-altering and exonic groupfiles. The default settings were used for kernel (linear.weighted), imputation methods (bestguess), number of resampling (2 × 10^6^) and p-value calculation (hybrid). Only genes with at least two variants were retained and multiple testing correction was performed using Bonferroni correction. Plots were generated with the R-package CMplot. An example of the gene-based test pipeline is available in Script File S1. Sanger sequencing was performed to confirm the presence of the variants in the top- and suggestive genes of the different gene-based tests in the cCSC patients (P < 5 × 10^−5^). Additionally, Burden, SKAT and SKAT-O p-values of previously associated cCSC genes and genes in their 100 kb up- and downstream region were extracted from the dataset.

The number of rare variant carriers (MAF < 0.05) in the four identified cCSC candidate genes was counted per gene in female and male cCSC patients and controls. The carrier and non-carrier groups were compared between controls and cCSC patients using a Chi-square test or Fisher’s exact test when at least 25% of the cells had an expected count <5. Odds ratios for single variants in the candidate genes were calculated using the Firth corrected likelihood method in SAS (v9.4)^19^, when variants were significantly associated with cCSC, because the SKAT single variant analysis only provides P-values and no risk estimates. Haplotypes in the *LAMB3* gene were constructed with the haplo.stats R-package as described before^10,20^.

### Ethics approval and consent to participate

This study was performed according to the guidelines of the Declaration of Helsinki and was approved by the local ethics committee. Written informed consent was obtained for all subjects involved in the study.

## Results

### Quality control

In this study, 269 cCSC patients and 1586 controls were analysed by exome sequencing. Among these samples 232 controls and six cCSC patients were not of European descent, and were therefore excluded from downstream analysis. Additionally, three control samples were removed due to cryptic relatedness (n = 2) or uncertain sex based on the X-chromosome data (n = 1). After individual quality control the dataset contained 263 cCSC patients (208 males [79%]) and 1352 controls (671 males [50%]). For these individuals, joint genotyping with GATK resulted in a dataset containing 746,264 SNPs and 45,048 INDELs. Of these 3,793 SNPs and 1,661 INDELs were multiallelic, and were split. Additional quality control included HWE filtering (P = 1 × 10^−8^) and removal of the low complexity regions of the genome^15^. Finally, a total of 579,602 variants remained for downstream analysis, of which 547,595 were SNPs and 32,007 were INDELS.

### Unbiased single variant association and gene-based analyses

Single variant association analysis corrected for sex and two principal components did not identify any genome-wide significant hits in the entire datasets, nor in the datasets stratified by sex (Fig. S3 in Document S1). To increase power to detect associations, gene-based analyses using the Burden, SKAT and SKAT-O tests were performed. These analyses were carried out on the entire cohort and on male and female cohorts stratified by sex, testing either all protein-altering variants or using all exonic variants. The number of genes and variants included in the gene-based analyses is described in Table 1.

**Table 1:** Number of variants and genes included in the gene-based analyses.

	Protein-altering variants^#^			Exonic variants^#^		
	Variants (n)	Genes (n)	Bonferroni correction p-value threshold	Variants (n)	Genes (n)	Bonferroni correction p-value threshold
All individuals	197,915	16,370	3.05 × 10^−6^	330,689	18,173	2.75 × 10^−6^
Males	135,468	15,083	3.31 × 10^−6^	232,667	17,348	2.88 × 10^−6^
Females	123,898	14,935	3.35 × 10^−6^	215,244	17,370	2.88 × 10^−6^

^#^Only genes with more than 2 variants were retained and used for Bonferroni correction.

**Figure 1:**
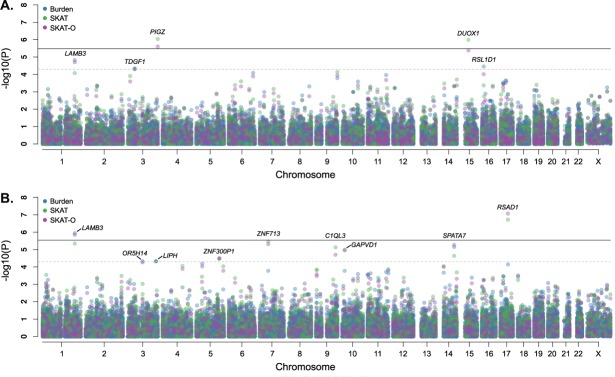
Manhattan plot of gene-based analysis in female cCSC patients. Burden (blue), SKAT (green) and SKAT-O (purple) association results are depicted for the female cohort including 55 cCSC patients vs. 681 controls, using the protein-altering (**A**) and exonic (**B**) group files. The dark horizontal line indicates the significance threshold after correction for multiple testing: 0.05/17,370 genes = 2.88 × 10^−6^ (**A**), 0.05/14,935 genes = 3.35 × 10^−6^ (**B**), while the dotted line indicates the suggestive threshold of P = 5 × 10^−5^.

In the protein-altering variant analysis, genome-wide significant associations with the *PIGZ* (P_SKAT_ = 9.19 × 10^−7^ & P_SKAT-O_ = 2.48 × 10^−6^) and *DUOX1* (P_SKAT_ = 1.03 × 10^−6^) genes were identified in females (Table 2, Fig. 1A). Additionally, suggestive associations in the protein-altering analysis (P < 5 × 10^−5^) were identified in the entire cohort, as well as the male and female cohort for *NOP14*, *RSPO2*, *LAMB3, TDGF1* and *RSL1D1* (Fig. 1A, Table S2 and Fig. S4 in Document S1). Gene-based analyses of the exonic variants identified genome-wide significant associations in the *RSAD1* (P_SKAT_ = 1.92 × 10^−7^ & P_SKAT-O_ = 8.57 × 10^−8^) and *LAMB3* (P_Burden_ = 1.40 × 10^−6^ & P_SKAT-O_ = 1.14 × 10^−6^) genes in the female cohort (Table 2, Fig. 1B). Gene-based analysis of the exonic variant on the entire cohort and the male cohort did not identify any genome-wide associations. Suggestive hits for the entire cohort and the stratified male and female cohorts were identified in *ALX1, NOP14, G3BP1*, *RNF144A, C5orf63, NOB1, LOC283332, ZNF713, SPATA7, C1QL3, GAPVD1, ZNF300P1, LIPH* and *OR5H14* (Fig. 1B, Table S2 and Fig. S4 in Document S1).

**Table 2:** Significant gene-based association results for the female cCSC cohort using the Burden, SKAT and SKAT-O tests.

Gene	Variants	Burden p-value	SKAT p-value	SKAT-O p-value
*PIGZ*	Protein-altering	6.99 × 10^−3^	9.19 × 10^−7**#**^	2.48 × 10^−6**#**^
*DUOX1*	Protein-altering	7.18 × 10^−3^	1.03 × 10^−6**#**^	4.23 × 10^−6^
*RSAD1*	Exonic	7.14 × 10^−5^	1.92 × 10^−7^*	8.57 × 10^−8^*
*LAMB3*	Exonic	1.40 × 10^−6^*	4.51 × 10^−6^	1.14 × 10^−6^*

^#^Significant after Bonferroni correction for 14,935 genes; P ≤ 3.35 × 10^−6^.

*Significant after Bonferroni correction for 17,370 genes; P ≤ 2.88 × 10^−6^.

### Rare variant carrier frequency in PIGZ, DUOX1, LAMB3 and RSAD1 in cCSC patients and controls

Gene-based analyses identified four cCSC-associated candidate genes in the female cohort. An overview of all variants found in *PIGZ, DUOX1, LAMB3* and *RSAD1* in females is presented in Table S2. The *PIGZ* gene-based association was explained by 14 rare (MAF < 0.05) and 4 common (MAF > 0.05) nonsynonymous variants. The percentage of rare variant carriers was higher in the female cCSC patient group compared to the female controls (Table 3, P = 0.014, Odds ratio (OR) = 3.67 [95% confidence interval (CI) = 1.42–9.47]). The p.A124V variant in *PIGZ* was individually associated with cCSC in females (P = 0.0003, OR = 16.40 [95% CI = 3.64–73.94]) (Table S2).

**Table 3:** Female carriers of rare variants (MAF < 0.05) in *PIGZ, DUOX1, LAMB3* and *RSAD1*.

Gene	Nr. rare variants in gene	Nr. carriers in controls (n_total_ = 681)	Nr. carriers in cCSC patients (n_total_ = 55)	P-value	OR (95% CI)
*PIGZ*	14	22 (3.2%)	6 (10.9%)	0.014	3.67 (1.42–9.47)
*DUOX1*	13	14 (2.1%)	4 (7.3%)	0.039	3.74 (1.19–11.77)
*LAMB3*	46	93 (13.7%)	17 (30.9%)	5.57 × 10^–4^	2.83 (1.53–5.22)
*RSAD1*	8	5 (0.7%)	3 (5.5%)	0.017	7.80 (1.81–33.55)
Variant in 1 or more genes		126 (18.5%)	25 (45.5%)	1.92 × 10^−6^	3.67 (2.09–6.46)

Nr. number; P-values are calculated with Chi-square test or Fisher’s exact when at least 25% of the cells had an expected count <5.

The *DUOX1* gene-based association was composed of 12 rare and 2 common nonsynonymous variants and 1 rare stop-gain variant. Of the rare variants, 3 were found in the cCSC group, whereas 11 were found in the control group. The percentage of carriers was significantly higher in the cCSC group compared to controls (Table 3, P = 0.039, OR = 3.74 [95% CI = 1.19–11.77]) The p.R925W variant was individually associated with cCSC (P = 0.03, OR = 62.69 [95% CI = 1.52− > 999.99]), and was only found in the female cCSC group (Table S2).

In the *LAMB3* gene, a total of 49 variants was observed in females. These variants included 31 nonsynonymous, 13 synonymous, 3 intronic variants near splice sites and 2 stop-gain variants. Three of the synonymous variants were common, while the remaining 46 variants were rare. In the control group 93 individuals (13.7%) carried at least one *LAMB3* rare variant, while 17 (30.9%) female cCSC patients carried one or more rare variants in *LAMB3* (Table 3, P = 0.00056, OR = 2.83 [95% CI = 1.53–5.22]). Haplotype analysis identified a significant association with cCSC for a haplotype carrying 7 variants in *LAMB3* (p.S97S, p.N181D, p.V527M, p.Y588Y, p.S708S, p.N690S, p.A926D) which had a frequency of 7.3% in the cCSC patients and 1.6% in the control cohort (Table S3, P = 7.22 × 10^−5^, OR = 6.60 [95% CI = 2.61–16.67]).

A total of 11 variants was present in *RSAD1* in females, of these 9 were nonsynonymous (7 rare, 2 common) variants, 1 was a common synonymous variant, and 1 was a rare intronic variant. The percentage of rare variant carriers was higher among female cCSC patients, compared to the control group (Table 3, P = 0.017, OR = 7.80 [95% CI = 1.81–33.55]). Three variants were only observed in the patient group (p.P250L, p.V299I, p.R317Q).

In total, 18.5% of the female control individuals carried at least one rare variant in *PIGZ, DUOX1, LAMB3* or *RSAD1*, whereas this percentage was 45.5% in the female cCSC patients. The percentage of rare variant carriers in these genes was significantly higher in the female cCSC patients group compared the control group (Table 3, P = 1.92 × 10^−6^, OR = 3.67 [95% CI = 2.09–6.46]). In males, no differences in the percentage of carriers of *PIGZ, DUOX1, LAMB3* or *RSAD1* rare variants were observed between controls and cCSC patients (Table S4). Also, the total percentage of carriers of at least one variant in these 4 genes was not different in males (Table S4).

### Gene-based association of genes located in known cCSC loci

Previous candidate gene and genome-wide association studies identified common, mostly non-coding variants in or near several genes in cCSC. We aimed to determine whether rare coding variants in these genes are associated with cCSC, since such associations could point towards casual genes in the known cCSC loci. Genes at previously associated cCSC loci and genes within ±100 kb of the top-associated variant were extracted from the dataset including the *ARMS2-HTRA1, C4-RCCX, CD46, CDH5, KCNT2-CFH-CFHR1/4, NR3C2, TNFRS10A* and *VIPR2* loci. In total, 56 genes were located in these regions, of which 39 genes contained at least 2 protein-altering variants and 44 genes had at least 2 variants in the exonic regions. The gene-based association results of these genes were extracted from the genome-wide analysis. However, no associations were identified between these genes and cCSC after correction for multiple testing (Table S5, Threshold P < 0.00114 [corrected for 44 genes in the exonic variant analysis] and P < 0.00128 [corrected for 39 genes in the protein-altering variant analysis]), indicating that rare coding variants at the known cCSC loci are not associated with cCSC in this cohort.

## Discussion

In this study, in order to increase our knowledge and understanding of the genetics of cCSC, we performed exome sequencing on a cohort of cCSC patients. Using a gene-based approach, we identified four potential new candidate genes for the disease in female cCSC patients specifically.

Due to a relative small sample size this study had limited power to identify single-variant associations, especially for rare variants, therefore, three different gene-based analyses with varying assumptions were performed. Previous studies have shown that the *CFH* gene carried both protective and risk-conferring variants^5,6^, therefore, we used the SKAT test, which assumes variants within a gene can have both directions of effect. The Burden test on the other hand relies on the assumption that all variants in a gene have the same direction of effect, and the SKAT-O test combines both assumptions into one test^18^. The gene-based analyses were performed using two different inclusion criteria. One analysis contained protein-altering variants only, since they are most likely to affect protein function. The other analysis contained all exonic variants, including synonymous (silent) variants and splice site variants, of which the effect on protein function is more difficult to predict. Nevertheless, such variants may alter gene expression levels and were therefore included in the analysis^21^. In the female cohort we identified a significant burden of protein-altering variants in two genes (*PIGZ* and *DUOX1*) and a significant burden of exonic variants in two additional genes (*LAMB3* and *RSAD1*).

Nearly half of the females in the cCSC cohort carried at least one variant in the four identified cCSC candidate genes (45.5%), while this number was significantly lower in the female control cohort (18.5%, P = 1.92 × 10^−6^). Carrying a rare variant in one of these genes increases the risk for developing cCSC 3.7-fold in females, while carrying a variant in *RSAD1* even increases risk 7.8-fold. Notably, a number of high-risk variants were identified in the gene-based tests, including the p.A124V variant in *PIGZ* (P = 0.0003, OR = 16.40 [95% CI = 3.64–73.94]) and the p.R925W variant in the *DUOX1* gene (P = 0.03, OR = 62.69 [95% CI = 1.52− > 999.99]), and three variants (p.P250L, p.V299I, p.R317Q) in *RSAD1* were observed only in the patient group but not in the controls. The percentage of carriers of rare variants in the *PIGZ, DUOX1, LAMB3* and *RSAD1* genes was not different between male cCSC patients and controls (Table S4, 15.9% vs. 20.4%, respectively, P = 0.146), indicating that the observed associations with cCSC are sex-specific. Since in our exome sequencing dataset of 263 patients only 55 were female, replication in an independent female cCSC cohort is necessary to substantiate these findings.

Interestingly, genome-wide significant associations were only observed in female cCSC patients but not in the males. The enrichment of rare variants in these genes in females is likely not due to population stratification, sequencing artefacts or the presence of related individuals, because stringent quality control was performed to avoid these confounders. It remains unclear why no associations in the male cCSC cohort were observed, despite accounting for the largest proportion of the cohort. One could hypothesize that cCSC is more genetically heterogeneous in males, and that a larger sample size is therefore necessary to identify associations in this group. The biological mechanism behind these sex-specific associations remains to be elucidated, but sex-biased gene expression or regulation could be one of the contributing factors^22,23^. Interactions between sex and genetics have been described for other complex traits such as hypertension, schizophrenia and juvenile idiopathic arthritis^23–25^, and for cCSC the previously described *CDH5* association was observed in males only^7^. Future genetic studies on cCSC, should focus on increasing samples size, but also should take sex-stratification into account, to further elucidate a possible interaction between sex and genetics.

For cCSC specifically, an interaction between genetic variants and sex hormone regulation and expression seems likely, since the association of cCSC with the stress axis, hypercortisolism and the use of corticosteroids has been described extensively, and pregnancy is known to increase the risk of CSC in females^1,26^. An interaction with the steroid hormone system has already been described for *LAMB3* and *DUOX1*^27,28^. For the *LAMB3* gene, Zhao *et al*. described upregulation of gene expression in endometrial biopsies after stimulation of 17β-estradiol and progesterone, both hormones involved mainly in the female reproductive system^27^; for the *DUOX1* gene, Ko *et al*. described increased expression after testosterone stimulation in keratinocytes^28^. Future studies might study the effect of variants in these genes on their response to hormonal differences in cellular models. Additionally, all four identified candidate genes are expressed in different tissues of the eye including the retina and RPE (Fig. S5 in Document S1)^29^. In light of the cCSC phenotype, for all four genes the effect of the identified variants on the disease mechanisms remains to be determined. Additionally, future experiments could focus on determining if a steroid hormone response is also present in the retina, RPE and/or choroid for these genes, and if the identified rare variants might alter this response.

## Conclusions

In this exome sequencing study on a cohort of cCSC patients and population controls, we identified *PIGZ, DUOX1, LAMB3* and *RSAD1* as potential new candidate genes for cCSC in females specifically. Our results suggest that the contribution of rare genetic risk variants might be different between the sexes and that the cCSC disease mechanism might differ between males and females. Replication of the current findings in larger cCSC cohorts is necessary, and further genetic studies and clinical trials on cCSC could take an interaction component between sex and genetics into account for cCSC.

## Supplementary information

Supplementary files

## Data Availability

The datasets used and/or analysed during the current study are available from the corresponding author on reasonable request.
