# Supplementary material for: Exome sequencing in patients with chronic central serous chorioretinopathy
Source: Sci Rep. 2019 Apr 29;9:6598. doi: 10.1038/s41598-019-43152-3 (PMC6488596; doi:10.1038/s41598-019-43152-3)
Supplement: Supplementary file 1 — Supplementary files [file 41598_2019_43152_MOESM1_ESM.pdf]

Exome sequencing in patients with chronic central serous chorioretinopathy

Rosa L. Schellevis[1], Myrte B. Breukink[1], Christian Gilissen[2], Camiel J.F. Boon[3], Carel B. Hoyng[1], Eiko K. de Jong*[1] & Anneke I. den Hollander*[1, 2]

[1] Department of Ophthalmology, Donders Institute of Brain, Cognition, and Behaviour, *Radboud University Medical Center*, Nijmegen, the Netherlands

[2] Department of Genetics, Donders Institute of Brain, Cognition, and Behaviour, *Radboud University Medical Center*, Nijmegen, the Netherlands

[3] Department of Ophthalmology, *Leiden University Medical Center*, Leiden, the Netherlands

* Authors contributed equally to the work

Supplementary files

- Figure S1: Principal component analysis (PCA) of the cCSC patients and controls.
- Figure S2: Kinship analysis of cCSC patients and controls
- Figure S3: Single variant analysis of the exome sequencing results in cCSC patients and controls.
- Figure S4: Gene-based analysis in the entire cCSC cohort and the male cCSC cohort
- Figure S5: Gene expression of *DUOX1, LAMB3, PIGZ* and *RSAD1* in different tissues
- Table S1: Suggestive gene-based associations using the Burden, SKAT and SKAT-O tests
- Table S2: Variants observed in the *DUOX1, PIGZ, LAMB3* and *RSAD1* genes in the female cohort
- Table S3**:** Haplotypes of *LAMB3* in the female cohort
- Table S4: Male rare variant (maf<0.05) carriers in *PIGZ, DUOX1, LAMB3* and *RSAD1*
- Table S5: Gene-based results of genes in the known cCSC loci
- Script File S1**:** R commands performed to run the Burden, SKAT, SKAT-O and single variant analysis.

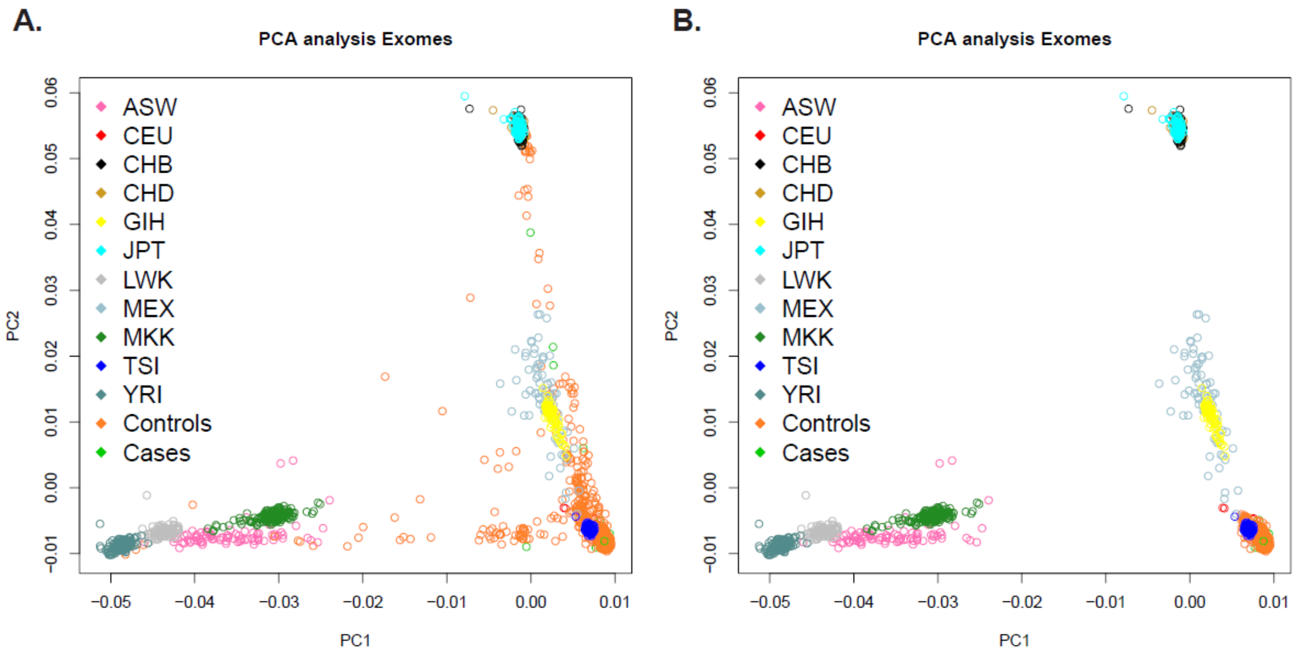

Figure S1. Principal component analysis (PCA) of the cCSC patients and controls. Graphical representation of the first 2 principal components of the PCA analysis, before (A) and after (B) filtering based on European ancestry. Various colours indicate the Hapmap3 samples according to the legend, orange depicts the controls and light green depicts the cCSC patients.

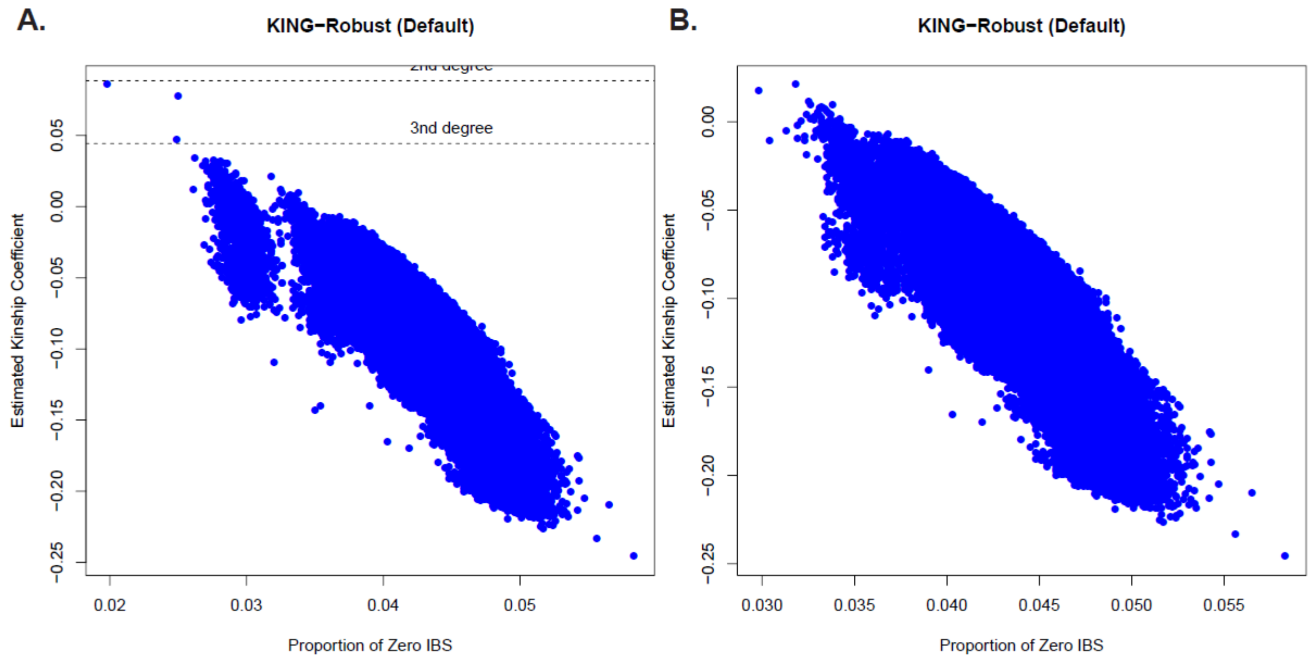

Figure S2. Kinship analysis of cCSC patients and controls. Kinship analysis was performed with KING, and estimated kinship coefficients of individuals in the study were calculated (A). Individuals with a kinship > 0.0442 were removed from the analysis (B).

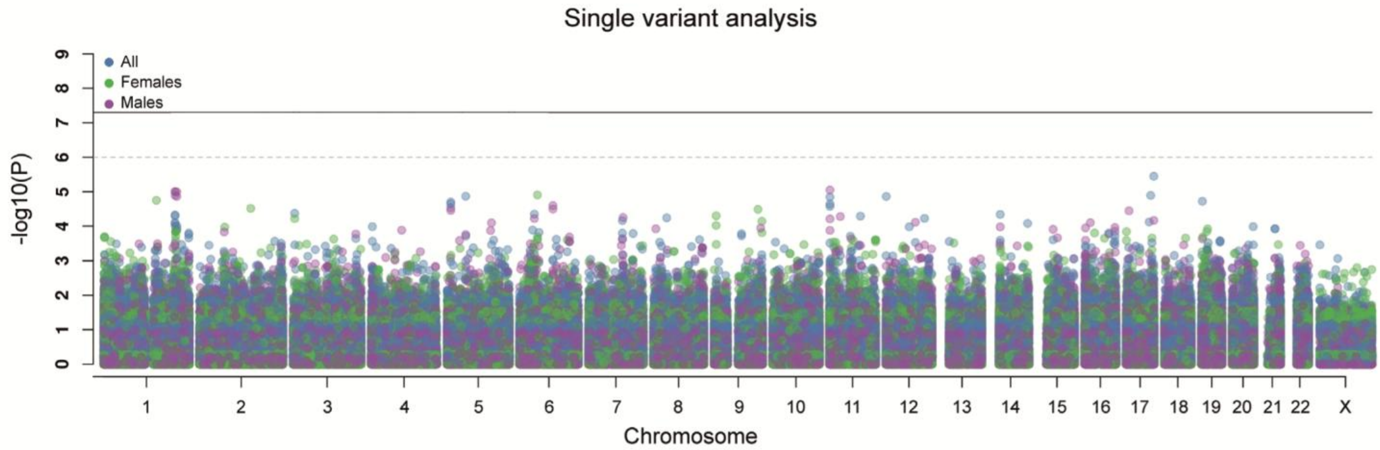

Figure S3. Single variant analysis of the exome sequencing results in cCSC patients and controls. Single variant analysis was performed with correction for 2 principal components and gender in the complete cohort (263 cCSC patients vs. 1352 controls [blue]), while for the females (55 cCSC patients vs. 681 controls [green]) and males (208 cCSC patients vs. 671 controls [purple]) only correction with the 2 principal components was applied. The dark horizontal line indicates the significance threshold after correction for multiple testing of $5x10^{-8}$, while the dotted line indicates the suggestive threshold of $P=1x10^{-6}$.

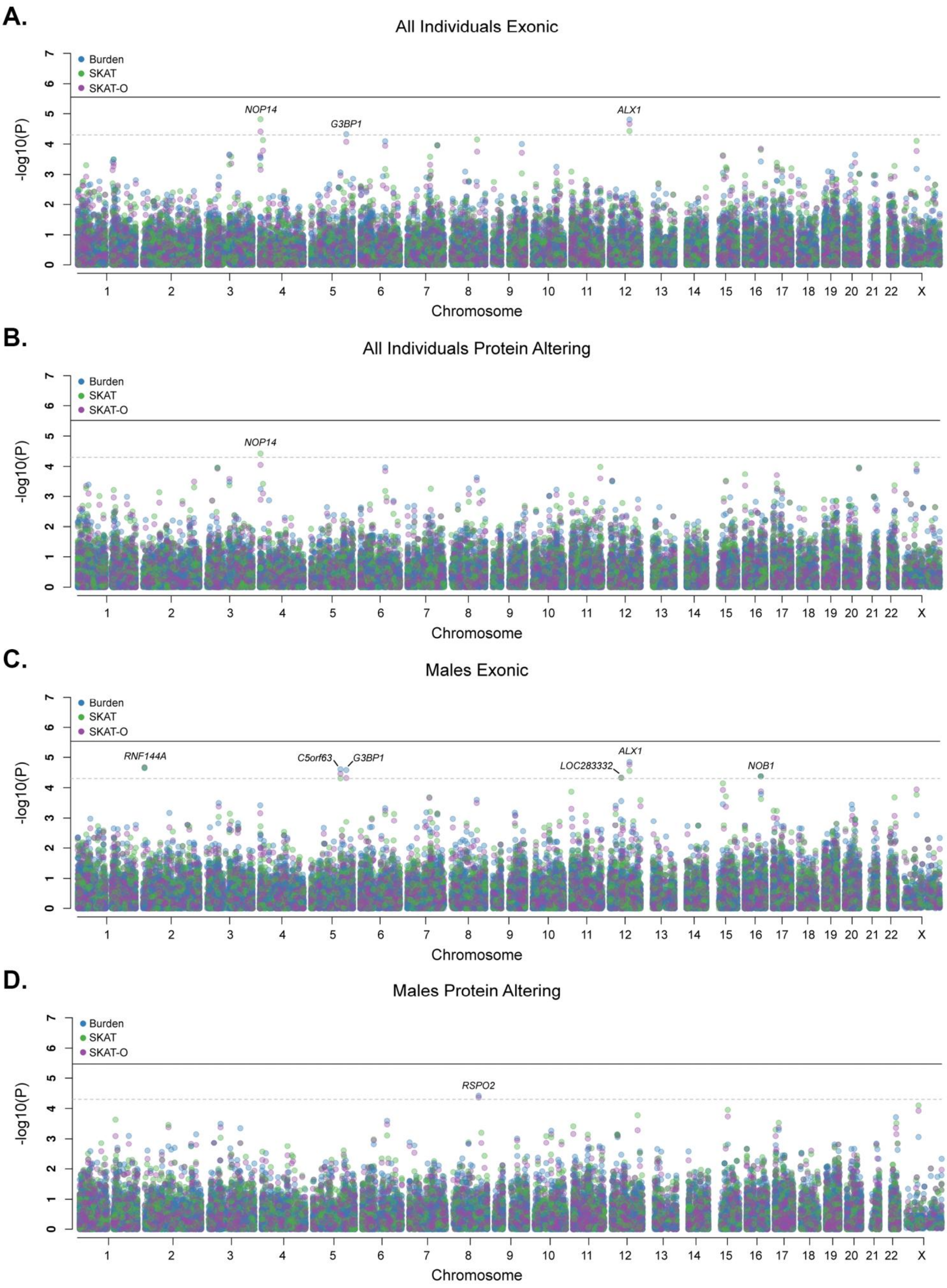

Figure S4. Gene-burden analysis all cCSC individuals and the male cCSC cohort.

Burden (blue), SKAT (green) and SKAT-O (purple) association results are depicted for the complete cohort of 263 cCSC patients and 1352 controls corrected for gender and 2 principal components, using the Exonic (A) and Protein Altering (B) group files. The results the gene-burden test in the 208 male cCSC patients and 671 male controls, for both group files are also depicted (C+D). The dark horizontal line indicates the significance threshold after correction for multiple testing: 0.05/18,173 genes=$2.75x10^{-6}$ (A), 0.05/16,370 genes=$3.05x10^{-6}$ (B), 0.05/17,348 genes=$2.88x10^{-6}$ (C) and 0.05/15,083 genes=$3.31x10^{-6}$ (D), while the dotted line indicates the suggestive threshold of P=$5x10^{-5}$.

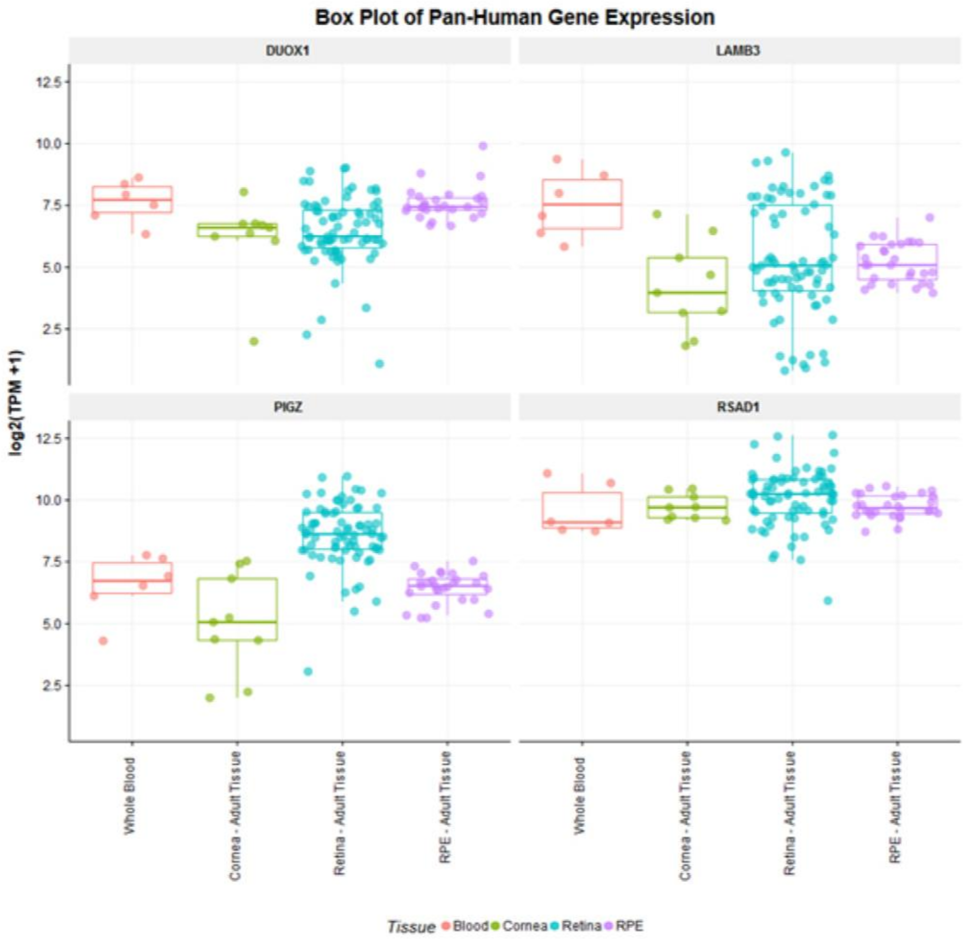

Figure S5. Gene expression of *DUOX1, LAMB3, PIGZ* and *RSAD1* in different tissues.
Pan-Human Gene Expression levels of the four susceptibility genes in females according to the eyeintegration database v0.63 (27). Whole blood (red), Cornea (green), retina (blue) and retinal pigment epithelium (RPE, purple) expression levels are depicted.

Table S1. Suggestive gene-based associations using the Burden, SKAT and SKAT-O tests				
Gene	***Variants***	***Burden p-value***	***SKAT p-value***	***SKAT-O p-value***
All individuals				
ALX1	Exonic	$1.57x10^{-5}$	$3.71x10^{-5}$	$2.17x10^{-5}$
NOP14	Protein-altering/ Exonic	$3.02x10^{-2}$/ $2.49x10^{-2}$	$3.76x10^{-5}$/ $1.52x10^{-5}$	$8.88x10^{-5}$/ $3.88x10^{-5}$
G3BP1	Exonic	$4.72x10^{-5}$	$1.71x10^{-3}$	$8.45\ x10^{-5}$
Males				
RSPO2	Protein-altering	$3.81x10^{-5}$	$1.95x10^{-2}$	$4.37x10^{-5}$
ALX1	Exonic	$1.43x10^{-5}$	$2.80x10^{-5}$	$1.74x10^{-5}$
RNF144A	Exonic	$2.25x10^{-5}$	$2.25x10^{-5}$	$2.12x10^{-5}$
C5orf63	Exonic	$2.48x10^{-5}$	$4.92x10^{-5}$	$3.49x10^{-5}$
NOB1	Exonic	$4.24x10^{-5}$	$4.24x10^{-5}$	$4.24x10^{-5}$
LOC283332	Exonic	$2.77x10^{-4}$	$4.81x10^{-5}$	$4.64x10^{-5}$
G3BP1	Exonic	$2.63x10^{-5}$	$1.36x10^{-3}$	$4.81x10^{-5}$
Females				
ZNF713	Exonic	$1.68x10^{-4}$	$3.40x10^{-6}$	$4.86x10^{-6}$
SPATA7	Exonic	$7.11x10^{-6}$	$2.30x10^{-5}$	$5.35x10^{-6}$
C1QL3	Exonic	$1.68x10^{-4}$	$1.03x10^{-5}$	$1.10x10^{-5}$
GAPVD1	Exonic	$1.45x10^{-2}$	$7.43x10^{-6}$	$1.99x10^{-5}$
ZNF300P1	Exonic	$3.33x10^{-5}$	$3.33x10^{-5}$	$3.16x10^{-5}$
LIPH	Exonic	$4.73x10^{-5}$	$4.73x10^{-5}$	$4.64x10^{-5}$
TDGF1	Protein-altering	$4.89x10^{-5}$	$4.89x10^{-5}$	$4.64x10^{-5}$
OR5H14	Exonic	$4.67\ x10^{-5}$	$9.33x10^{-4}$	$5.42x10^{-5}$
RSL1D1	Protein-altering	$3.52x10^{-5}$	$4.28x10^{-4}$	$9.66x10^{-5}$

Table S4. Male rare variant (MAF <0.05) carriers in *PIGZ*, *DUOX1*, *LAMB3* and *RSAD1*					
	No. rare variants in gene	Controls (n=671)	cCSC patients (n=208)	P-value	OR (95% CI)
LAMB3	32	81 (12.1%)	20 (9.6%)	0.38	0.78 (0.46-1.30)
PIGZ	19	35 (5.2%)	7 (3.4%)	0.35	0.63 (0.28-1.45)
DUOX1	23	24 (3.6%)	8 (3.8%)	1.00	1.08 (0.48-2.44)
RSAD1	4	7 (1.0%)	1 (0.5%)	0.69	0.46 (0.06-3.73)
Variant in 1 or more genes		137 (20.4%)	33 (15.9%)	0.15	0.74 (0.49-1.12)

Table S2. Variants observed in the *DUOX1, PIGZ, LAMB3* and *RSAD1* genes in the female cohort

Gene	Variant	Exon	Effect	Nucleotide change	Protein change	SKAT P-value	SAS P-value	OR (95% CI)	Weight	Counts female cCSC (n=55)	Counts female controls (n=681)	MAF cCSC (n=55)	MAF controls	Exac_NFE	PhyloP	Grantham	Mutation Taster (score)	CADD	Sift (score)	Polyphen (score)	Trap(score)
DUOX1 (NM_175940)	rs764911806	5	nonsyn	c.C411G	p.D137E	0.50	0.54	4.09 (0.04-381.40)	1.00E+00	0	1	0	7.34E-04	1.00E-04	0.25	45	N(1)	15.8	T (0.361)	B (0.036)	0.147
	rs200421952	14	nonsyn	c.G1627A	p.A543T	0.55	0.64	2.46 (0.06-102.04)	1.00E+00	0	2	0	1.47E-03	7.00E-04	1.61	58	N(0.953)	13.6	T (0.159)	B (0.124)	0.001
	rs201198610	15	nonsyn	c.T1778C	p.F593S	0.54	0.54	4.09 (0.04-381.40)	1.00E+00	0	1	0	7.34E-04	6.00E-04	1.29	155	D(0.558)	20.9	T (0.45)	B (0.138)	0.012
	rs370633829	15	nonsyn	c.G1786A	p.G596R	**0.044**	0.12	37.16 (0.40->999.99)	1.00E+00	1	0	9.09E-03	0	7.50E-05	1.53	125	N(0.643)	24.4	T (0.068)	D (0.999)	0.234
	rs143304688	17	stopgain	c.C1948T	p.Q650*	0.55	0.54	4.09 (0.04-381.40)	1.00E+00	0	1	0	7.34E-04	1.70E-03	.	.	A(1)	44.0	.	.	0.773
	rs368978619	18	nonsyn	c.T2152A	p.L718M	0.57	0.64	2.46 (0.06-102.04)	1.00E+00	0	2	0	1.47E-03	2.00E-04	0.06	15	N(1)	8.1	T (0.266)	B (0.003)	0.004
	rs373898472	21	nonsyn	c.G2750T	p.W917L	0.54	0.54	4.09 (0.04-381.40)	1.00E+00	0	1	0	7.34E-04	3.00E-05	3.56	61	D(1)	17.0	T (0.831)	D (0.957)	0.021
	rs145668427	21	nonsyn	c.C2773T	p.R925W	**0.0024**	**0.03**	62.69 (1.52->999.99)	1.00E+00	2	0	0.018	0	2.40E-03	0.99	101	D(1)	34.0	D (0.004)	D (1)	0.048
	rs148902727	21	nonsyn	c.C2794T	p.R932C	0.57	0.64	2.46 (0.06-102.04)	1.00E+00	0	2	0	1.47E-03	5.00E-04	3.23	180	D(1)	26.6	D (0.014)	D (1)	0.038
	rs16939752	24	nonsyn	c.T3076C	p.C1026R	0.33	0.30	1.35 (0.77-2.37)	1.09E-03	15	146	0.136	0.107	0.127	0.98	180	P(0.899)	15.4	T (0.364)	B (0)	0.026
	rs777590867	24	nonsyn	c.G3170C	p.G1057A	0.051	0.08	12.41 (0.77-199.80)	1.00E+00	1	1	0.01	7.34E-04	1.52E-05	1.46	60	D(1)	10.9	T (0.38)	Prob. D (0.661)	0.014
	rs2458236	27	nonsyn	c.C3532T	p.L1178F	0.99	0.91	0.98 (0.65-1.47)	2.24E-17	37	467	0.336	0.343	0.674	0.85	22	P(0.999)	20.5	T (0.439)	B (0.002)	0.09
	rs142004175	28	nonsyn	c.G3644A	p.R1215H	0.56	0.54	4.09 (0.04-381.40)	1.00E+00	0	1	0	7.34E-04	1.60E-03	3.38	29	D(1)	33.0	D (0.002)	D (1)	0.059
	rs138894830	28	nonsyn	c.G3656A	p.R1219Q	0.51	0.54	4.09 (0.04-381.40)	1.00E+00	0	1	0	7.34E-04	1.30E-03	3.38	43	D(1)	23.8	T (0.277)	Prob. D (0.706)	0.332
	rs773444369	33	nonsyn	c.C4462T	p.R1488C	0.55	0.54	4.09 (0.04-381.40)	1.00E+00	0	1	0	7.34E-04	4.50E-05	2.20	180	D(1)	33.0	D (0)	D (1)	0.116
PIGZ (NM_025163)	rs74554271	2	nonsyn	c.C65T	p.P22L	0.54	0.54	4.09 (0.04-381.40)	1.00E+00	0	1	0	7.34E-04	2.00E-04	0.26	98	N(1)	16.23	D (0.015)	B (0.002)	0.021
	rs780890452	3	nonsyn	c.C308T	p.T103I	0.53	0.54	4.09 (0.04-381.40)	1.00E+00	0	1	0	7.34E-04	9.98E-05	2.40	89	D(1)	15.80	D (0.018)	B (0.036)	0.214
	rs200901195	3	nonsyn	c.G323A	p.R108K	0.53	0.54	4.09 (0.04-381.40)	1.00E+00	0	1	0	7.34E-04	2.00E-04	0.29	26	D(1)	9.08	T (1)	B (0)	0.475
	rs199852771	3	nonsyn	c.C344T	p.P115L	0.60	0.75	1.76 (0.06-54.12)	1.00E+00	0	3	0	2.20E-03	4.00E-04	-0.93	98	N(1)	0.00	T (0.407)	B (0)	0.123
	rs144088027	3	nonsyn	c.C371T	p.A124V	**0.00046**	**0.00030**	16.40 (3.64-73.94)	1.00E+00	4	3	0.036	2.20E-03	5.70E-03	-1.40	64	N(1)	0.00	T (1)	B (0.002)	0.144
	rs150001262	3	nonsyn	c.G464A	p.R155H	0.53	0.54	4.09 (0.04-381.40)	1.00E+00	0	1	0	7.34E-04	1.30E-03	0.66	29	D(1)	23.20	T (0.129)	D (1)	0.128
	rs148186466	3	nonsyn	c.G725T	p.G242V	0.63	0.85	1.364 (0.052-36.04)	1.00E+00	0	4	0	2.94E-03	2.00E-04	0.78	109	D(0.821)	16.08	T (0.164)	Prob. D (0.934)	0.327
	rs574365	3	nonsyn	c.G796A	p.A266T	0.58	0.56	0.87 (0.55-1.39)	2.11E-12	24	334	0.218	0.245	0.744	0.20	58	P(1)	0.27	T (0.323)	B (0.001)	0.063
	rs140946995	3	nonsyn	c.C821T	p.A274V	0.57	0.64	2.46 (0.06-102.04)	1.00E+00	0	2	0	1.47E-03	1.50E-03	1.61	64	N(1)	0.00	T (1)	B (0.136)	0.064
	rs148546943	3	nonsyn	c.C864A	p.N288K	0.54	0.54	4.09 (0.04-381.40)	1.00E+00	0	1	0	7.34E-04	1.57E-04	0.52	94	.(.)	0.03	T (0.49)	B (0.002)	0.011
	rs149631795	3	nonsyn	c.C1010T	p.A337V	0.55	0.54	4.09 (0.04-381.40)	1.00E+00	0	1	0	7.34E-04	4.00E-04	0.76	64	N(1)	7.99	T (0.164)	Prob. D (0.919)	0.078
	rs4916589	3	nonsyn	c.G1019A	p.R340Q	0.75	0.72	0.93 (0.63-1.38)	9.96E-23	43	557	0.391	0.409	0.596	0.46	43	P(1)	8.52	T (0.505)	B (0)	0.166
	rs150517093	3	nonsyn	c.C1078T	p.R360W	0.54	0.54	4.09 (0.04-381.40)	1.00E+00	0	1	0	7.34E-04	4.00E-04	-2.76	101	N(1)	0.00	T (0.188)	B (0.001)	0.009
	rs1147238	3	nonsyn	c.T1250C	p.V417A	0.47	0.42	1.24 (0.73-2.09)	3.51E-05	18	189	0.164	0.139	0.141	0.98	64	P(0.947)	7.25	T (0.252)	B (0.007)	0.081
	rs150414171	3	nonsyn	c.G1401C	p.M467I	0.11	0.09	7.45 (0.71-77.77)	1.00E+00	1	2	9.09E-03	1.47E-03	1.90E-03	3.82	10	D(0.937)	25.20	D (0.036)	D (1)	0.043
	rs17855662	3	nonsyn	c.G1461A	p.M487I	0.14	0.19	0.55 (0.23-1.33)	8.97E-02	5	117	0.045	0.086	0.084	2.20	10	N(0.712)	22.10	T (0.143)	Prob. D (0.524)	0.431
	rs202156268	3	nonsyn	c.G1466A	p.G489E	0.50	0.54	4.09 (0.04-381.40)	1.00E+00	0	1	0	7.34E-04	3.00E-05	3.82	98	D(0.931)	24.90	D (0.005)	D (0.998)	0.297
	rs146219795	3	nonsyn	c.G1591A	p.V531M	**0.042**	0.12	37.16 (0.40->999.99)	1.00E+00	1	0	9.09E-03	0	5.00E-04	-0.60	21	N(1)	2.73	T (0.18)	Prob. D (0.539)	0.01

	rs140470350	1	syn	c.C15T	p.F5F	0.53	0.54	4.09 (0.04-381.40)	1.00E+00	0	1	0	7.34E-04	2.60E-03	.	.	.	.	.	.	0.096
	rs2228339	*2*	*syn*	*c.C138T*	*p.T46T*	*0.77*	0.76	1.07 (0.68-1.70)	*2.84E-11*	*26*	*308*	*0.236*	0.226	*0.245*	.	.	.	.	.	.	0.044
	rs2076356	*3*	*syn*	*c.A291C*	*p.S97S*	*0.06*	0.07	1.44 (0.97-2.14)	*2.06E-18*	*46*	*454*	*0.418*	0.333	*0.659*	.	.	.	.	.	.	0.023
	rs2235542	5	nsyn	c.A541G	p.N181D	**0.00015**	**<0.0001**	5.11 (2.33-11.22)	9.99E-01	9	24	8.18E-02	1.76E-02	2.26E-02	0.953	23	P(1)	5.937	T(0.461)	B(0.036)	0
	rs150895872	5	nsyn	c.G548A	p.R183H	0.57	0.54	4.09 (0.04-381.40)	1.00E+00	0	1	0	7.34E-04	1.40E-03	0.015	29	N(1)	12.49	T(0.282)	B(0.004)	0.054
	rs121912482	6	nsyn	c.G628A	p.E210K	0.55	0.54	4.09 (0.04-381.40)	1.00E+00	0	1	0	7.34E-04	.	1.461	56	A(0.922)	22.5	T(0.398)	B(0.238)	0.988
	rs141059189	8	syn	c.C927T	p.D309D	0.51	0.54	4.09 (0.04-381.40)	1.00E+00	0	1	0	7.34E-04	3.00E-04	.	.	.	.	.	.	0.028
	rs886045867	8	syn	c.A943C	p.R315R	0.53	0.54	4.09 (0.04-381.40)	1.00E+00	0	1	0	7.34E-04		.	.	.	.	.	.	0.875
	rs52814161	9	nsyn	c.T1015C	p.Y339H	0.15	0.15	2.37 (0.72-7.75)	1.00E+00	3	18	2.73E-02	1.32E-02	1.72E-02	1.190	83	N(1)	7.697	T(0.287)	B(0)	0.008
	rs114875539	9	nsyn	c.G1051A	p.E351K	0.57	0.64	2.46 (0.06-102.04)	1.00E+00	0	2	0	1.47E-03	1.60E-03	1.145	56	N(1)	17.25	T(0.333)	D(0.998)	0
	rs548096567	9	nsyn	c.C1069T	p.R357W	**0.037**	0.12	37.16 (0.40->999.99)	1.00E+00	1	0	9.09E-03	0	0	0.351	101	N(1)	33	D(0.004)	D(1)	0.157
	rs2076351	10	syn	c.G1149A	p.P383P	0.69	0.90	0.82 (0.04-17.55)	1.00E+00	0	7	0	5.14E-03	5.90E-03	.	.	.	.	.	.	0.003
	rs779219301	10	nsyn	c.G1189A	p.G397R	0.52	0.54	4.09 (0.04-381.40)	1.00E+00	0	1	0	7.34E-04	5.38E-05	5.261	125	D(1)	29.4	D(0.001)	D(1)	0.38
	1:209801457	10	nsyn	c.A1211G	p.H404R	0.53	0.54	4.09 (0.04-381.40)	1.00E+00	0	1	0	7.34E-04	.	4.536	29	N(0.859)	13.61	D(0.017)	B(0.101)	0.307
	rs2229468	11	nsyn	c.T1312A	p.S438T	0.54	0.54	4.09 (0.04-381.40)	1.00E+00	0	1	0	7.34E-04	3.00E-04	-0.062	58	P(1)	4.981	T(1)	B(0.002)	0.056
	rs762400142	11	syn	c.G1347A	p.G449G	0.54	0.54	4.09 (0.04-381.40)	1.00E+00	0	1	0	7.34E-04	0	.	.	.	.	.	.	0.018
	1:209800257	12	stgain	c.C1552T	p.Q518X	0.54	0.54	4.09 (0.04-381.40)	1.00E+00	0	1	0	7.34E-04		.	.	A(1)	38	.	.	0.015
	rs771727419	12	nsyn	c.G1574C	p.G525A	0.54	0.54	4.09 (0.04-381.40)	1.00E+00	0	1	0	7.34E-04	1.55E-05	5.575	60	D(1)	24.9	D(0.008)	D(1)	0.102
	1:209800233	12	nsyn	c.G1576A	p.D526N	0.56	0.54	4.09 (0.04-381.40)	1.00E+00	0	1	0	7.34E-04		1.497	23	N(0.98)	17.93	D(0.016)	D(0.974)	0.005
	rs2076349	12	nsyn	c.G1579A	p.V527M	**0.00091**	**0.0005**	3.83 (1.79-8.19)	9.99E-01	9	32	8.18E-02	2.35E-02	2.76E-02	-0.266	21	P(1)	10.99	T(0.068)	P(0.596)	0.007
	rs767004520	13	nsyn	c.C1654T	p.R552C	**0.031**	0.12	37.16 (0.40->999.99)	1.00E+00	1	0	9.09E-03	0	1.00E-04	2.557	180	D(0.999)	34	D(0.006)	D(1)	0.02
	rs2179402	*13*	*syn*	*c.T1716C*	*p.C572C*	*0.19*	0.19	1.32 (0.87-1.99)	*2.94E-14*	*36*	*369*	*0.327*	0.271	*0.747*	.	.	.	.	.	.	0.014
	rs370280436	13	nsyn	c.G1721A	p.R574H	0.57	0.64	2.46 (0.06-102.04)	1.00E+00	0	2	0	1.47E-03	7.64E-05	0.516	29	N(0.978)	21.9	T(0.069)	B(0.039)	0.002
	rs771720883	13	syn	c.G1728A	p.P576P	0.52	0.54	4.09 (0.04-381.40)	1.00E+00	0	1	0	7.34E-04	1.00E-04	.	.	.	.	.	.	0.024
LAMB3 (NM_001017402)	rs2229465	13	syn	c.T1764C	p.Y588Y	**0.00019**	**<.0001**	4.91 (2.25-10.74)	9.99E-01	9	25	8.18E-02	1.84E-02	2.45E-02	.	.	.	.	.	.	0.002
	rs760667887	13	nsyn	c.G1853A	p.R618H	0.54	0.54	4.09 (0.04-381.40)	1.00E+00	0	1	0	7.34E-04	3.01E-05	-4.245	29	N(1)	0.066	T(0.574)	B(0)	0
	rs80356682	13	stgain	c.C1903T	p.R635X	0.14	0.09	7.45 (0.71-77.77)	1.00E+00	1	2	9.09E-03	1.47E-03	1.30E-03	.	.	A(1)	36	.	.	0.007
	1:209799027	13	nsyn	c.G1942A	p.V648M	0.55	0.54	4.09 (0.04-381.40)	1.00E+00	0	1	0	7.34E-04	.	0.301	21	N(0.976)	21.2	T(0.052)	B(0.048)	0.017
	1:209799011	13	nsyn	c.G1958T	p.S653I	0.50	0.54	4.09 (0.04-381.40)	1.00E+00	0	1	0	7.34E-04	.	0.199	142	N(1)	24.6	D(0.005)	P(0.641)	0.019
	rs2229466	14	nsyn	c.A2069G	p.N690S	**0.00014**	**<0.0001**	5.11 (2.33-11.22)	9.99E-01	9	24	8.18E-02	1.76E-02	2.21E-02	0.225	46	P(1)	0.003	T(0.243)	B(0.003)	0.271
	rs2072937	14	syn	c.T2124C	p.S708S	**0.00014**	**<0.0001**	5.11 (2.33-11.22)	9.99E-01	9	24	8.18E-02	1.76E-02	2.21E-02	.	.	.	.	.	.	0.041
	rs778247685	15	nsyn	c.C2209T	p.R737C	**0.039**	0.12	37.16 (0.40->999.99)	1.00E+00	1	0	9.09E-03	0	1.51E-05	-0.134	180	N(1)	16.04	T(0.061)	P(0.876)	0.052
	rs113745536	15	syn	c.G2298A	p.K766K	0.57	0.64	2.46 (0.06-102.04)	1.00E+00	0	2	0	1.47E-03	8.00E-04	.	.	.	.	.	.	0.005
	1:209796424	16	nsyn	c.G2459A	p.G820D	0.52	0.54	4.09 (0.04-381.40)	1.00E+00	0	1	0	7.34E-04	.	4.023	94	D(1)	29.5	D(0.004)	D(1)	0.088
	rs151006337	17	nsyn	c.C2564G	p.A855G	0.55	0.64	2.46 (0.06-102.04)	1.00E+00	0	2	0	1.47E-03	1.00E-04	0.396	60	N(1)	4.838	T(0.074)	B(0.039)	0.01
	rs539752226	17	nsyn	c.T2588G	p.I863S	0.57	0.64	2.46 (0.06-102.04)	1.00E+00	0	2	0	1.47E-03	.	2.704	142	N(0.842)	24.7	D(0.001)	P(0.808)	0.452
	rs199946321	17	nsyn	c.C2632T	p.R878C	0.53	0.54	4.09 (0.04-381.40)	1.00E+00	0	1	0	7.34E-04	7.49E-05	1.177	180	D(0.999)	34	D(0)	D(1)	0.049
	rs2076222	18	nsyn	c.C2777A	p.A926D	**0.00014**	**<0.0001**	5.11 (2.33-11.22)	9.99E-01	9	24	8.18E-02	1.76E-02	2.21E-02	3.355	126	P(0.06)	24.5	D(0.002)	D(0.999)	0.001
	rs775023132	18	nsyn	c.T2813C	p.I938T	0.56	0.54	4.09 (0.04-381.40)	1.00E+00	0	1	0	7.34E-04	1.50E-05	2.485	89	D(0.605)	26.5	D(0.021)	D(0.979)	0.035
	rs777389254	18	nsyn	c.G2833A	p.V945M	0.54	0.54	4.09 (0.04-381.40)	1.00E+00	0	1	0	7.34E-04	1.50E-05	1.167	21	N(0.986)	27	D(0.049)	D(0.999)	0.032
	rs143164025	18	syn	c.G2871A	p.A957A	0.63	0.75	1.76 (0.06-54.12)	1.00E+00	0	3	0	2.20E-03	2.00E-04	.	.	.	.	.	.	0.031
	rs140769823	19	nsyn	c.A2945G	p.D982G	0.59	0.75	1.76 (0.06-54.12)	1.00E+00	0	3	0	2.20E-03	1.60E-03	2.079	94	N(0.857)	17.12	T(0.574)	B(0)	0.578
	1:209791343	19	nsyn	c.T2960G	p.L987R	0.53	0.54	4.09 (0.04-381.40)	1.00E+00	0	1	0	7.34E-04	.	3.490	102	D(0.942)	29	D(0.006)	P(0.821)	0.017
	rs147527725	20	nsyn	c.G3068A	p.R1023Q	0.07	0.08	12.41 (0.77-199.80)	1.00E+00	1	1	9.09E-03	7.34E-04	3.00E-04	0.063	43	N(1)	18.77	T(0.55)	B(0.244)	0.171
	rs147931502	22	nsyn	c.A3479G	p.H1160R	0.52	0.54	4.09 (0.04-381.40)	1.00E+00	0	1	0	7.34E-04	7.00E-04	1.698	29	N(0.998)	12.25	T(0.119)	B(0.201)	0.097
	rs745444340	22	nsyn	c.G3487A	p.G1163R	0.53	0.54	4.09 (0.04-381.40)	1.00E+00	0	1	0	7.34E-04	3.00E-05	-0.135	125	N(1)	0.188	T(0.677)	B(0.003)	0.032
	rs901375494	Intr15	.	G>A	0	0.50	0.54	4.09 (0.04-381.40)	1.00E+00	0	1	0	7.34E-04	.	.	.	.	.	.	.	0.053
	rs757259426	Intr2	.	G>A	0	**0.048**	0.12	37.16 (0.40->999.99)	1.00E+00	1	0	9.09E-03	0	0.00E+00	.	.	.	.	.	.	0.065
	rs375846575	Intr6	.	G>C	0	0.56	0.54	4.09 (0.04-381.40)	1.00E+00	0	1	0	7.34E-04	6.02E-05	.	.	.	.	.	.	0.38
	rs2290862	*3*	*nsyn*	*c.G355A*	*p.A119T*	*0.55*	0.70	0.93 (0.62-1.38)	*1.66E-22*	*41*	*534*	*0.373*	0.392	*0.613*	*-0.041*	*58*	*P(1)*	*8.627*	*T(0.446)*	*B(0)*	0.09
	rs2290861	*3*	*nsyn*	*c.T377C*	*p.L126S*	*0.55*	0.70	0.93 (0.62-1.38)	*1.66E-22*	*41*	*534*	*0.373*	0.392	*0.614*	*4.026*	*145*	*P(1)*	*18.49*	*T(0.455)*	*B(0)*	0.304
	17:48557374	3	nsyn	c.A403C	p.T135P	0.54	0.54	4.09 (0.04-381.40)	1.00E+00	0	1	0	7.34E-04	.	0.959	38	D(0.975)	23	D(0.002)	D(0.999)	0.066
	rs201488851	4	nsyn	c.C512T	p.T171M	0.50	0.54	4.09 (0.04-381.40)	1.00E+00	0	1	0	7.34E-04	2.00E-04	1.188	81	N(0.983)	24	T(0.105)	D(0.996)	0.002
RSAD1 (NM_018346)	*rs7210579*	*4*	*syn*	*c.A582G*	*p.V194V*	*0.55*	0.62	1.12 (0.72-1.75)	*4.12E-11*	*28*	*321*	*0.255*	0.236	*0.243*	.	.	.	.	.	.	0.083
	rs137932823	4	nsyn	c.G745C	p.D249H	0.53	0.54	4.09 (0.04-381.40)	1.00E+00	0	1	0	7.34E-04	1.00E-03	3.211	81	D(0.939)	24.3	D(0.003)	P(0.74)	0.02
	rs541139014	4	nsyn	c.C749T	p.P250L	**0.031**	0.12	37.16 (0.40->999.99)	1.00E+00	1	0	9.09E-03	0	4.60E-05	1.761	98	D(0.999)	19.12	T(0.059)	P(0.895)	0.21
	rs143685560	5	nsyn	c.G895A	p.V299I	**0.021**	0.12	37.16 (0.40->999.99)	1.00E+00	1	0	9.09E-03	0	2.00E-04	0.130	29	N(1)	9.465	T(1)	B(0.028)	0.099
	rs778299565	6	nsyn	c.G950A	p.R317Q	**0.039**	0.12	37.16 (0.40->999.99)	1.00E+00	1	0	9.09E-03	0	4.62E-05	3.475	43	D(0.997)	26.6	D(0.038)	D(0.992)	0.281
	rs374609288	7	nsyn	c.C1072G	p.L358V	0.52	0.54	4.09 (0.04-381.40)	1.00E+00	0	1	0	7.34E-04	2.00E-04	1.226	32	N(0.997)	22.9	D(0.007)	B(0.201)	0.079
	17:48561127	0	.	G>A	0	0.53	0.54	4.09 (0.04-381.40)	1.00E+00	0	1	0	7.34E-04	.	.	.	.	.	.	.	0.236

Table S3. Haplotypes of *LAMB3* in the female cohort

Corrected with PC1 and PC2

Haplotype	rs745444340	rs147931502	rs147527725	1:209791343	rs140769823	rs143164025	rs777389254	rs775023132	rs2076222	rs199946321	rs539752226	rs151006337	1:209796424	rs901375494	rs113745536	rs778247685	rs2072937	rs2229466	1:209799011	1:209799027	rs80356682	rs760667887	rs2229465	rs771720883	rs370280436	rs2179402	rs767004520	rs2076349	1:209800233	rs771727419	1:209800257	rs762400142	rs2229468	1:209801457	rs779219301	rs2076351	rs548096567	rs114875539	rs52814161	rs886045867	rs141059189	rs375846575	rs121912482	rs150895872	rs2235542	rs2076356	rs757259426	rs2228339	rs140470350	Pooled HF	Control HF	cCSC patients HF	P-value	OR (95% CI)
1	0	0	0	0	0	0	0	0	0	0	0	0	0	0	0	0	0	0	0	0	0	0	0	0	0	0	0	0	0	0	0	0	0	0	0	0	0	0	0	0	0	0	0	0	0	0	0	0	0	0.508	0.518	0.412	NA	Base
5	0	0	0	0	0	0	0	0	0	0	0	0	0	0	0	0	0	0	0	0	0	0	0	0	0	0	0	0	0	0	0	0	0	0	0	0	0	0	0	0	0	0	0	0	0	1	0	0	0	0.012	0.013	NA	0	0 (0-0)
6	0	0	0	0	0	0	0	0	0	0	0	0	0	0	0	0	0	0	0	0	0	0	0	0	0	0	0	0	0	0	0	0	0	0	0	0	0	0	0	0	0	0	0	0	0	1	0	1	0	0.143	0.145	0.129	0.929	1.03 (0.53-2.00)
23	0	0	0	0	0	0	0	0	0	0	0	0	0	0	0	0	0	0	0	0	0	0	0	0	0	1	0	0	0	0	0	0	0	0	0	0	0	0	0	0	0	0	0	0	0	1	0	1	0	0.062	0.060	0.053	0.447	1.40 (0.59-3.30)
20	0	0	0	0	0	0	0	0	0	0	0	0	0	0	0	0	0	0	0	0	0	0	0	0	0	1	0	0	0	0	0	0	0	0	0	0	0	0	0	0	0	0	0	0	0	0	0	0	0	0.116	0.114	0.116	0.521	1.24 (0.64-2.41)
11	0	0	0	0	0	0	0	0	0	0	0	0	0	0	0	0	0	0	0	0	0	0	0	0	0	0	0	0	0	0	0	0	0	0	0	0	0	0	1	0	0	0	0	0	0	0	0	0	0	0.014	0.012	NA	0.108	2.72 (0.80-9.18)
22	0	0	0	0	0	0	0	0	0	0	0	0	0	0	0	0	0	0	0	0	0	0	0	0	0	1	0	0	0	0	0	0	0	0	0	0	0	0	0	0	0	0	0	0	0	1	0	0	0	0.077	0.074	0.112	0.045	2.03 (1.02-4.04)
55	0	0	0	0	0	0	0	0	1	0	0	0	0	0	0	0	1	1	0	0	0	0	1	0	0	0	0	1	0	0	0	0	0	0	0	0	0	0	0	0	0	0	0	0	1	1	0	0	0	0.020	0.016	0.073	7.22E-05	6.60 (2.61-16.67)

Base: Haplotype used as reference in the association model; HF: Haplotype frequency; NA: Not annotated; PC: Principal component of the ancestry analysis

Table S5. Gene-burden results of genes in the known cCSC loci

		All individuals								Males								Females							
		exonic				protein altering				exonic				protein altering				exonic				protein altering			
Gene	Locus	Variants	Burden	SKAT	SKAT-O	Variants	Burden	SKAT	SKAT-O	Variants	Burden	SKAT	SKAT-O	Variants	Burden	SKAT	SKAT-O	Variants	Burden	SKAT	SKAT-O	Variants	Burden	SKAT	SKAT-O
ARMS2	*ARMS2-HTRA1*	*6*	0.358	0.681	0.498	5	0.593	0.865	0.725	5	0.377	0.602	0.500	4	0.635	0.633	0.636	4	0.784	0.781	1.000	4	0.784	0.781	1.000
HTRA1	*ARMS2-HTRA1*	*10*	0.763	0.910	0.918	6	0.844	0.632	0.866	9	0.716	0.859	0.887	5	0.885	0.470	0.637	4	0.807	0.571	0.913	2	0.634	0.634	0.634
PLEKHA1	*ARMS2-HTRA1*	*18*	0.572	0.267	0.432	12	0.118	0.248	0.201	15	0.209	0.149	0.254	10	0.020	0.096	0.036	11	0.157	1.000	0.274	9	0.204	0.854	0.349
CR1L	*CD46*	*8*	0.542	0.844	0.645	7	0.546	0.877	0.663	7	0.673	1.000	0.779	6	0.671	1.000	0.775	7	0.689	0.801	0.797	6	0.673	0.879	0.773
CD46	*CD46*	*10*	0.405	0.689	0.504	7	0.347	0.666	0.431	7	0.163	0.151	0.195	5	0.131	0.147	0.165	7	0.373	0.050	0.072	6	0.389	0.049	0.071
CD34	*CD46*	*16*	0.219	0.033	0.047	9	0.113	0.033	0.047	12	1.000	0.575	0.712	7	0.795	0.539	0.667	10	0.0091	0.0016	0.0026	6	0.0067	0.0017	0.0027
BEAN1	*CDH5*	*24*	0.758	1.000	0.892	16	0.583	0.668	0.694	16	0.589	0.633	0.756	9	0.525	0.594	0.635	17	1.000	0.390	0.554	10	1.000	1.000	1.000
CDH5	*CDH5*	*39*	0.787	0.892	1.000	23	0.867	0.577	0.798	25	0.549	0.596	0.721	15	0.321	0.264	0.434	29	0.122	1.000	0.247	17	0.126	0.982	0.228
CFHR2	*CFH*	*6*	0.090	0.389	0.136	4	0.193	0.494	0.270	6	0.249	0.685	0.342	4	0.326	0.729	0.432	4	0.128	0.507	0.193	3	0.424	0.678	0.590
CFHR4	*CFH*	*7*	0.294	0.146	0.242	4	0.262	0.709	0.400	6	1.000	0.410	0.607	4	0.255	0.709	0.391	2	0.069	0.069	0.071	NA	NA	NA	NA
KCNT2	*CFH*	*26*	0.733	0.188	0.271	11	0.140	0.057	0.119	14	0.552	0.278	0.384	4	0.033	0.130	0.100	17	0.086	0.204	0.135	8	0.957	0.539	0.957
CFHR5	*CFH*	*24*	0.374	0.358	0.524	19	0.350	0.338	0.497	17	0.943	0.641	0.813	15	0.952	0.613	0.787	17	0.085	0.050	0.091	13	0.064	0.047	0.080
CFH	*CFH*	*33*	0.717	0.333	0.515	19	0.276	0.464	0.423	24	0.184	0.073	0.126	14	0.059	0.128	0.099	23	0.182	0.924	0.315	13	0.254	0.562	0.373
NR3C2	*NR3C2*	*15*	0.227	0.737	0.348	8	0.109	0.782	0.185	12	0.332	0.881	0.485	5	0.159	0.783	0.233	14	0.465	0.593	0.641	7	0.379	0.677	0.581
ARHGAP10	*NR3C2*	*31*	0.570	0.630	0.742	16	0.202	0.932	0.276	24	0.866	0.363	0.523	12	0.282	1.000	0.378	18	0.419	1.000	0.590	7	0.422	1.000	0.548
PPT2	*RCCX*	*4*	0.170	0.109	0.151	3	0.789	0.793	1.000	4	0.128	0.143	0.190	3	0.454	0.455	0.455	2	0.210	0.210	0.210	2	0.210	0.210	0.210
STK19	*RCCX*	*10*	0.590	0.600	0.754	4	0.187	0.519	0.380	8	0.846	0.965	0.966	3	0.638	0.763	0.741	5	0.058	0.210	0.149	3	0.105	0.294	0.294
ZBTB12	*RCCX*	*8*	0.482	0.617	0.566	5	0.372	0.776	0.481	5	0.516	0.599	0.608	2	0.343	0.721	0.721	5	1.000	1.000	1.000	3	0.603	0.603	0.603
FKBPL	*RCCX*	*7*	0.699	0.729	0.852	6	0.889	0.746	0.885	7	0.740	0.264	0.394	6	1.000	0.283	0.416	6	1.000	0.289	0.433	5	1.000	0.270	0.406
RDBP	*RCCX*	*10*	0.040	0.018	0.040	8	0.046	0.021	0.028	7	0.035	0.011	0.020	5	0.012	0.010	0.017	7	0.541	0.490	0.710	5	0.718	0.906	0.906
DOM3Z	*RCCX*	*19*	1.000	0.777	1.000	9	1.000	0.783	1.000	15	1.000	0.502	0.673	8	0.600	0.478	0.586	13	1.000	0.443	0.607	5	0.526	0.299	0.371
EGFL8	*RCCX*	*14*	0.655	0.223	0.332	10	0.064	0.162	0.107	10	1.000	0.374	0.495	7	0.119	0.201	0.194	10	0.320	0.323	0.426	7	0.184	1.000	0.298
CFB	*RCCX*	*17*	0.350	0.703	0.505	11	0.204	0.646	0.310	15	0.836	1.000	1.000	9	0.891	0.895	1.000	13	0.117	0.101	0.149	9	0.0060	0.056	0.012
ATF6B	*RCCX*	*21*	0.173	0.682	0.268	11	0.248	1.000	0.373	16	0.522	1.000	0.668	8	0.402	0.808	0.578	13	0.070	0.124	0.116	9	0.569	0.804	0.835
SLC44A4	*RCCX*	*25*	0.483	0.641	0.656	15	0.404	0.706	0.597	14	0.257	1.000	0.431	10	0.348	0.837	0.527	21	1.000	0.611	0.774	12	0.870	0.863	1.000
EHMT2	*RCCX*	*36*	0.208	0.183	0.284	16	0.153	0.073	0.113	22	0.656	0.538	0.745	11	0.548	0.317	0.471	25	0.129	0.108	0.159	12	0.086	0.070	0.101
C2	*RCCX*	*24*	0.811	0.645	0.786	20	0.660	0.591	0.728	18	0.682	0.676	0.811	14	0.679	0.654	0.787	14	1.000	0.316	0.430	13	1.000	0.393	0.512
AGER	*RCCX*	*31*	0.209	0.745	0.324	20	0.275	0.368	0.385	25	0.320	0.818	0.475	15	0.397	0.521	0.535	19	0.384	0.582	0.545	11	0.396	0.713	0.509
SKIV2L	*RCCX*	*45*	0.922	0.283	0.429	24	0.798	0.215	0.327	33	0.596	0.050	0.080	19	0.343	0.077	0.125	30	0.447	0.420	0.601	14	0.167	0.273	0.262
NOTCH4	*RCCX*	*72*	0.685	1.000	0.856	47	0.544	0.939	0.740	55	0.591	1.000	0.769	32	0.756	0.695	0.863	49	0.093	0.220	0.150	29	0.074	0.448	0.128
TNXB	*RCCX*	*116*	0.519	0.404	0.576	84	0.221	0.346	0.351	87	0.228	0.048	0.085	63	0.048	0.050	0.066	71	0.394	0.159	0.251	52	0.284	0.141	0.238
GPSM3	*RCCX*	*2*	0.221	0.221	0.221	NA	NA	NA	NA	2	0.094	0.094	0.094	NA	NA	NA	NA	2	1.000	1.000	1.000	NA	NA	NA	NA
MIR1236	*RCCX*	*3*	0.104	0.379	0.196	NA	NA	NA	NA	2	0.076	0.279	0.123	NA	NA	NA	NA	1	NA	NA	NA	NA	NA	NA	NA
RNF5	*RCCX*	*3*	0.530	0.482	0.575	NA	NA	NA	NA	3	0.591	1.000	0.716	NA	NA	NA	NA	2	0.037	0.037	0.037	NA	NA	NA	NA
RNF5P1	*RCCX*	*3*	0.544	0.544	0.544	NA	NA	NA	NA	3	0.372	0.372	0.372	NA	NA	NA	NA	3	0.729	0.729	0.729	NA	NA	NA	NA
AGPAT1	*RCCX*	*4*	0.793	0.357	0.723	NA	NA	NA	NA	3	0.347	0.747	0.541	NA	NA	NA	NA	3	0.115	0.115	0.115	NA	NA	NA	NA
TNFRSF10C	*TNFRSF10A*	*7*	0.755	0.554	0.699	3	0.970	0.267	0.269	3	0.786	0.809	0.926	NA	NA	NA	NA	7	0.176	0.335	0.338	3	0.079	0.079	0.079
CHMP7	*TNFRSF10A*	*6*	0.544	0.791	0.815	6	0.544	0.791	0.815	4	0.527	0.753	0.787	4	0.527	0.753	0.787	2	0.585	0.585	0.585	2	0.585	0.585	0.585
R3HCC1	*TNFRSF10A*	*18*	0.936	0.829	1.000	8	0.674	0.980	0.779	15	0.836	0.687	0.813	6	0.632	1.000	0.736	13	0.785	1.000	1.000	6	0.934	1.000	1.000
TNFRSF10D	*TNFRSF10A*	*20*	0.483	0.030	0.051	10	0.330	0.505	0.454	12	0.786	0.114	0.181	8	0.317	0.535	0.439	18	0.337	0.179	0.275	8	0.823	0.782	1.000
TNFRSF10A	*TNFRSF10A*	*18*	0.893	0.049	0.089	15	0.271	0.017	0.030	14	0.575	0.119	0.203	12	0.890	0.057	0.100	12	0.250	0.193	0.317	10	0.097	0.118	0.143
LOXL2	*TNFRSF10A*	*36*	0.881	0.518	0.707	15	0.470	0.061	0.112	29	0.360	0.182	0.290	11	1.000	0.603	0.806	23	0.131	0.569	0.218	10	0.079	0.102	0.148
VIPR2	*VIPR2*	*19*	0.608	0.659	0.809	7	0.818	0.649	0.815	11	0.603	0.754	0.798	4	0.764	0.683	0.781	15	0.867	0.590	0.800	5	0.954	0.307	0.307
WDR60	*VIPR2*	*41*	0.499	0.282	0.415	27	0.547	0.843	0.733	29	0.417	0.355	0.512	20	0.396	0.817	0.569	22	0.979	0.596	0.754	13	1.000	0.495	0.674

Yellow color indicated P-values < 0.05. Correction for multiple testing was applied for 42 for the exonic groupfile (P<0.0119) and for 37 genes for the protein altering groupfile (P<0.001351). No genes were significantly associated after correction for multiple testing.

Script file S1

```
#Convert data to plink files and make a dummy gene list based on the
variants in the bim files
#In the following format:
#dummygene       SNP1
#dummygene       SNP2
awk '{print("dummygene""\t"$2)}' input.bim | sort -k2 -u >
SNPsets_dummy.txt

#Generate weight file based on minor allele frequency
R
library(SKAT)
#Generate the File.SSD and File.Info
Generate_SSD_SetID("input.bed", "input.bim", "input.fam",
"SNPsets_dummy.txt", "File.SSDdummy", "File.Infodummy")
#read the covariate file and fam file if applicable
FAM_Cov<-Read_Plink_FAM_Cov("input.fam", "input_cov.txt",
Is.binary=TRUE, flag1=0, cov_header=TRUE)
SSD.INFOdummy<-Open_SSD("File.SSDdummy", "File.Infodummy")
#Get genotype matrix of the dummy geneset
dummy_geneset<-Get_Genotypes_SSD(SSD.INFOdummy, 1, is_ID = TRUE)
weight<-Get_Logistic_Weights(dummy_geneset, par=0.07, par2=150)
weights<-data.frame(weight)
weights[,2] <- colnames(dummy_geneset)
weights = weights[, c(2,1)]
write.table(weights, file="snp_weights.txt", col.names=FALSE,
row.names=FALSE, sep="\t", quote = FALSE)

#perform single variant association with dummy geneset dataset
#Define the covariates if applicable
Phenotype = FAM_Cov$Phenotype
PC1 = FAM_Cov$PC1
PC2 = FAM_Cov$PC2
Sex = FAM_Cov$Sex
#Run the null model
obj<-SKAT_Null_Model_MomentAdjust(Phenotype ~ PC1 + PC2 + Sex,
type.Resampling="bootstrap.fast")
#Run the loop through the genotype matrix and output the p-values
single_pvalue <- NULL
for(snp in colnames(dummy_geneset)){out <-
SKATBinary_Single(dummy_geneset[,snp], obj); single_pvalue <-
rbind(single_pvalue, data.frame(snp, out$p.value, out$method.bin,
out$m))}
#print the output file
write.table(single_pvalue, file="Single_pvalue_all.txt",
col.names=TRUE, row.names=FALSE, sep="\t", quote = FALSE)

#Run the burden, SKAT and SKAT-O test using the snp_weights_file.txt
Generate_SSD_SetID("input.bed", "input.bim", "input.fam",
"groupfile.txt", "File.SSD_groupfile", "File.Info_groupfile")
#read the covariate file and fam file if applicable
FAM_Cov<-Read_Plink_FAM_Cov("input.fam", "input_cov.txt",
Is.binary=TRUE, flag1=0, cov_header=TRUE)
SSD.INFO_groupfile<-Open_SSD("File.SSD_groupfile",
"File.Info_groupfile")
Phenotype = FAM_Cov$Phenotype
PC1 = FAM_Cov$PC1
PC2 = FAM_Cov$PC2
Sex = FAM_Cov$Sex.x
obj<-SKAT_Null_Model_MomentAdjust(Phenotype ~ Sex + PC1 + PC2,
type.Resampling="bootstrap.fast")
custom_weights<-Read_SNP_WeightFile("snp_weights.txt")
```

```
#Burden test
out.burden<-SKATBinary.SSD.All(loaded_SSD, obj, method="Burden",
obj.SNPWeight=custom_weights, kernel="linear.weighted")
#SKAT test
out.skat<-SKATBinary.SSD.All(loaded_SSD, obj, method="SKAT",
obj.SNPWeight=custom_weights, kernel="linear.weighted")
#SKAT-O test
out.skato<-SKATBinary.SSD.All(loaded_SSD, obj, method="SKATO",
obj.SNPWeight=custom_weights, kernel="linear.weighted")

#keep only genes with more than 2 variants and perform correction
for multiple testing
out.burden_select <- out.burden[out.burden$N.Marker.Test>1,]
out.burden_select$FDR <- p.adjust(out.burden_select$P.value,
method="fdr")
out.burden_select$Bonf <- p.adjust(out.burden_select$P.value,
method="bonferroni")
write.table(out.burden_select, file="burden_output_groupfile.txt",
col.names=TRUE, row.names=FALSE, sep="\t", quote = FALSE)

out.skat_select <- out.skat[out.skat$N.Marker.Test>1,]
out.skat_select$FDR <- p.adjust(out.skat_select$P.value,
method="fdr")
out.skat_select$Bonf <- p.adjust(out.skat_select$P.value,
method="bonferroni")
write.table(out.skat_select, file="skat_output_groupfile.txt",
col.names=TRUE, row.names=FALSE, sep="\t", quote = FALSE)

out.skato_select <- out.skato[out.skato$N.Marker.Test>1,]
out.skato_select$FDR <- p.adjust(out.skato_select$P.value,
method="fdr")
out.skato_select$Bonf <- p.adjust(out.skato_select$P.value,
method="bonferroni")
write.table(out.skato_select, file="skato_output_groupfile.txt",
col.names=TRUE, row.names=FALSE, sep="\t", quote = FALSE)

#plot the data in CMplot
library(CMplot)
ref_genes=read.table("genes_location.txt", header=T)
data_combined1 <- merge(out.burden_select[,c(1,2)],
out.skat_select[,c(1,2)], by="SetID", all=TRUE)
data_combined2 <- merge(data_combined1, out.skato_select[,c(1,2)],
by="SetID", all=TRUE)
data_combined3 <- merge(ref_genes, data_combined2 , by=1,
no.dups=TRUE)
colnames(data_combined3) =
c("GENE","CHR","BP","burden","skat","skato")
nrow(data_combined3)
#genome-wide significant line --> 0.05/nrow=pvalue_cutoff
data_combined3[is.na(data_combined3)] <- 1

CMplot(data_combined3, plot.type="m", multracks=TRUE,
threshold=c(pvalue_cutoff,suggestive_line),threshold.lty=c(1,2),
        threshold.lwd=c(1,1), threshold.col=c("black","grey"),
amplify=TRUE,bin.size=1e6,
        chr.den.col=NULL,
signal.col=c("red","green"),signal.cex=c(1,1),
        file="pdf",memo="results")
```
